# Supplementary material for: Care practices, popular knowledge, and health promotion among quilombola woman in Brazil
Source: PLoS One. 2026 Feb 20;21(2):e0343298. doi: 10.1371/journal.pone.0343298 (PMC12922991; doi:10.1371/journal.pone.0343298)
Supplement: S1 File — (DOCX) [file pone.0343298.s001.docx]

# Supplementary File – Full Quotes and Interpretations

|  |  |
| --- | --- |
| We go for walks, we used to have Zumba, but we stopped more because of the alcoholism issue. But we used to have a lot of dance parties, but we stopped... so we take advantage of everything in this space. Walking is much healthier for us; with so many plants, we've learned not to burn them (Quilombola woman 2). | Physical activity and the natural environment form an accessible community health practice. |
| We have our walks, our work, all that busyness of doing things. We shouldn't be idle because it's bad to be idle (Quilombola woman 7). | Daily rural labor and walking maintain health and prevent idleness. |
| I walk in the morning, three times a week, to help me maintain my health and try to eat healthy things; we eat mostly fish (Quilombola woman 3). | Routine walking and natural diet as active health maintenance. |
| We do physical exercise, walking. Besides that, there's no other way for us to exercise. Promoting health is more about food, since it's very healthy (Quilombola woman 12). | Health defined through diet and walking. |
| My practice is that I am from the Umbanda religion, and in our religion we often say that whether you come with money or without money, the important thing is to do good, to help others... so we try to help not only the people of the community but also people from outside, even though it is a religion that is very criticized, with a lot of prejudice even today, but we always try to help and give our best. We offer spiritual treatments for psychological healing; sometimes a person arrives with an obsessive spirit that keeps disturbing them... I help some people who arrive, I can say, who are spiritually ill, I try to help (Quilombola Woman 12). | Spirituality functions as emotional, psychological and communal healing. |
| ...you arrive there in Estiva and we take very good care of the environmental issue. My grandmother, my grandmother was from African-based religions, you know what that is... she had a gift and she had knowledge of herbs, she prayed, she guided people, so she was from African-based religions, she had knowledge, but her knowledge was that she cleansed people (Quilombola Woman 1). | Ancestral Afro-Brazilian spirituality connected to environmental care. |
| In our spiritual work, we use a lot of elements of nature, herbal baths for cleansing if you are in pain. For a headache, a bath with leaves from a fragrant plant to relieve headaches, folk healing practices, this type of practice (Quilombola Woman 12). | Nature-based spiritual rituals form holistic healing. |
| This knowledge, transmitted from generation to generation, in our case through conversation and dialogue about the practice itself, we talk and then show what really happens in practice so that they can learn... The practice we have here in the community is very important because it is knowledge that is passed down from generation to generation, and we young people accept this agreement of knowledge (Quilombola woman 2). | Knowledge transmission happens orally and experientially. |
| It's about us effectively passing on knowledge from generation to generation, knowing and understanding your history, your roots, and using this knowledge to pass it on to them as a guarantee (Quilombola woman 1). | Intergenerational learning reinforces identity and cultural continuity. |
| The knowledge passed down from generation to generation; we try to transmit it to future generations. We can even transmit it if, for example, I have a child, and if we talk about it, they think it's nonsense (Quilombola woman 6). | New generations devalue traditional knowledge. |
| It had something else, oh my god, I forgot, now it was arueira, that thing that closed off these practices, they are not being passed on to the new generations, they are not being passed on as they should be (Quilombola woman 7). | Elders perceive loss of ancestral practices. |
| In the hospital, given the complexity, there are midwives here. When things don't work out here, they go to the main hospital where there's a midwife. There's an experienced midwife there... When there's no passage, she'll do a cesarean section. Until then, the baby stays with the midwife until it comes out naturally (Quilombola woman 3). | Midwives provided safe and trusted childbirth care. |
| It gave a sense of security: you do this, you do that, you do that, and then you give birth. I gave birth with midwives like that. My mother has delivered many babies, my sister knows how to deliver babies (Quilombola woman 1). | Midwifery represented embodied female knowledge. |
| There are no more midwives now, after she died my aunt, one of her daughters was also a midwife... all the midwives died, there are none left... (Quilombola woman 5). | Complete loss of traditional childbirth knowledge. |
| As they pass by, one teaches you tea in line, you're in line today for medicine, and they're handing out medicine. There are people from my time, some new ones, and one who taught me. There's the gapou strip, and they've already finished the sticks that need to be given for everything, it's all there, each one is teaching a tea, and I, at least since I had congestion, haven't taken injections ... (Woman quilombola 13). | Herbal remedies circulate socially as shared pharmacology. |
| We have an ambulance, people, we have an ambulance to take you, but if you're really in pain inside, then we make a tea to relieve it ... if it goes away with the tea, we drink the tea and stay there, if it gets worse we have to find a way outside, sometimes they come to do it in Mirinzal (Woman quilombola 11). | Traditional medicine is first response; biomedicine is second step. |
| Lemongrass tea is great as a calming agent and also very good for the stomach. There's also gardenia, which is also very good for the heart, and there are numerous others (Woman quilombola 2 ). | Ethnobotanical knowledge is broad and precise. |
| We take pharmacy medicines, as we call them, but we also have a tea for diabetes, we have a tea to lower blood pressure for those with hypertension, we have a tea for itchy skin... (Woman quilombola 2). | Herbal and biomedical treatments coexist. |
| When the pain is very old, we make tea from Santa Maria... (Woman quilombola 11). | Use of plants linked to ancestral indications. |
| Tea practices include various teas; there's a tea we make from neem leaves... (Quilombola woman 11). | Multiple teas address multiple symptoms. |
| Any issue like flu, cough, body aches, even if someone fractures a bone, there are remedies available, and regarding folk healers, we still have a young woman (Quilombola woman 8). | Home remedies integrate body and spiritual care. |
| We take our own precautions... teachings of the old woman that she left for us... (Quilombola woman 1). | Intergenerational continuity of home remedies. |
| They don't give out herbal remedies... they don't know (Quilombola woman 13). | Biomedical professionals lack knowledge about traditional practices. |
| I have, I give you, ma'am, you have, give me... wherever you arrive there's something to eat... there's chicken, there's ora-pro-nóbis... (Quilombola woman 8). | Reciprocity and solidarity sustain food security. |
| The land for us is the usufruct... it's my plant... we work with organic fertilizer... (Quilombola woman 1). | Organic farming tied to identity and territory. |
| We learned to do crop rotation... beans, coconut, cashew, Jussara, watermelon... (Quilombola Woman 2). | Agroecology provides continuity and sustainability. |
| We have many natural resources from the community that we extract food from; fishing, chickens, pigs... (Quilombola woman 12). | Extractivism sustains livelihood. |
| In our fishing cultures, everything left by them is our survival... cassava, fishing, buriti, açaí (Quilombola woman 6). | Ancestral extractive practices. |
| We have a government program... CONAB donates it to CRAS (Quilombola woman 2). | Public policy strengthens economic autonomy. |
| She has always worked very hard for the association... brings income, financial support, social services (Quilombola woman 2). | Women's leadership strengthens political rights. |
| All the benefits... water, septic tanks, bathrooms, electricity, roads, houses... through the association (Quilombola Woman 6). | Association sustains territorial legitimation. |
