# Supplementary material for: Care practices, popular knowledge, and health promotion among quilombola woman in Brazil
Source: PLoS One. 2026 Feb 20;21(2):e0343298. doi: 10.1371/journal.pone.0343298 (PMC12922991; doi:10.1371/journal.pone.0343298)
Supplement: Attachment — Submitted filename: RESPONSE TO REVIEWERS.docx [file pone.0343298.s003.docx]

**RESPONSE TO REVIEWERS**

**Manuscript title:** Care practices, popular knowledge, and health promotion among quilombola women in Brazil

Dear Editor and Reviewers,

We would like to thank the Editor and the Reviewers for their careful reading of the manuscript and for their valuable and constructive comments. We greatly appreciate the recognition of the relevance, rigor, and social importance of this study. All suggestions were carefully considered, and the manuscript was revised accordingly to improve methodological clarity, conceptual precision, and internal consistency. All modifications are highlighted in red in the revised manuscript.

**REVIEWER #1**

**General Comments**

The manuscript was revised to improve methodological transparency and conceptual coherence, particularly in the Materials and Methods and Discussion sections.

Major Comments

1. Redundancy in the description of the hermeneutic-dialectical approach
The two redundant paragraphs were condensed into a single, more concise paragraph to improve clarity and flow.

2. Insufficient description of the statistical dimension of IRAMUTEQ
The Methods section was expanded to explicitly describe the chi-square test used in the Descending Hierarchical Classification, clarify the meaning of the p-value, and explain how statistically significant associations supported qualitative interpretation.

3. Missing Table 1
Table 1 (Sociodemographic characterization) was included in the Results section, and the text was revised for consistency.

4. Exclusion criteria and stigmatizing language
The exclusion criteria were revised to avoid stigmatizing language, specifying cognitive or communicative limitations rather than mental health conditions.

**Minor Comments**

Duplicate references were corrected, conceptual precision regarding Minayo (2021) was improved, missing references were added, and the use of Habermas and Gadamer was refined.

**Final Remarks**

We sincerely thank the reviewers for their valuable contributions, which strengthened the manuscript.

Sincerely,

The Authors
